# Noninvasive respiratory support with high-flow nasal cannula in endoscopic surgery in a patient with *Legionella Pneumophila* pneumonia: a case report

**DOI:** 10.1186/s13741-024-00385-9

**Published:** 2024-04-17

**Authors:** Vincenzo Pota, Francesco Coppolino, Annamaria Auricchio, Francesca Cardella, Maurizio Del Prete, Antonio Scalvenzi, Pasquale Sansone, Maria Beatrice Passavanti, Maria Caterina Pace

**Affiliations:** 1 Anaesthesia, Intensive Care and Pain Medicine, Dept. of Women, Child, General and Specialistic Surgery, University of Campania “L. Vanvitelli,”, Naples, Italy; 2 Dept. of Translational Medical Sciences, University of Campania “L. Vanvitelli,”, Naples, Italy

## Introduction

*Legionella pneumophila* is one of the most important causes of respiratory distress in humans. More than 30% of hospital-acquired pneumonia is caused by *L. pneumophila*. Postoperative anastomotic leak after esophageal resection represents a serious surgical complication with significant morbidity and mortality. The prompt diagnosis and the initiation of therapy are essential to ameliorate the outcome. The positioning of an esophageal endoprosthesis offers a minimally invasive therapeutic approach regarding sepsis control and early oral feeding, which however is also associated with procedure-specific complications. Last year, we efforted a case anastomotic leakage after esophageal resection by positioning of endoprosthesis by endoscopic way. The patient was affected by *L. pneumophila* pneumonia. The main problem for the anesthesiologist in this case is to search an adequate and safe perioperatory management because of the acute respiratory failure due to *L. pneumophila*. The patient presented a P/F ratio between 100 and 200 mmHg configuring a picture of moderate ARDS according to the Berlin criteria. Approximately, 50% of postoperative pulmonary complications (PPCs) are attributable to the patient’s underlying condition, while the remaining 50% are related to the type of surgery and anesthetic management (Canet and Gallart 2013).

For this reason, it was decided to use a noninvasive ventilation mode, in this case with high-flow nasal cannula (HFNC), to avoid the risk of postoperative pulmonary complications, which significantly increase during an invasive ventilation mode that requires neuromuscular blockade. This modality of oxygenation and ventilation allowed also a direct access to the mouth in order to proceed to endoscopy. The risk factors that have suggested avoiding endotracheal intubation (ETI) in favor of high-flow oxygenation, according to the American College of Physicians, are as follows: advanced age of the patient, male gender, ASA classification > 3, and active respiratory infection with associated ARDS (Smetana et al. 2006). The high-flow nasal cannulas are an oxygenation device capable of providing a humidified flow of 60 L/min and a Fio2 up to 100%. Since ARDS falls under purely hypoxemic respiratory failure (type 1), the ability to ensure a high inspiratory fraction of bone without the need of ETI is an excellent solution to avoid PPCs. Periprocedural oxygenation with HFNC with sedation in spontaneous breath allowed us to guarantee adequate saturation in a patient with moderate ARDS.

## Case report

**Fig. 1:**
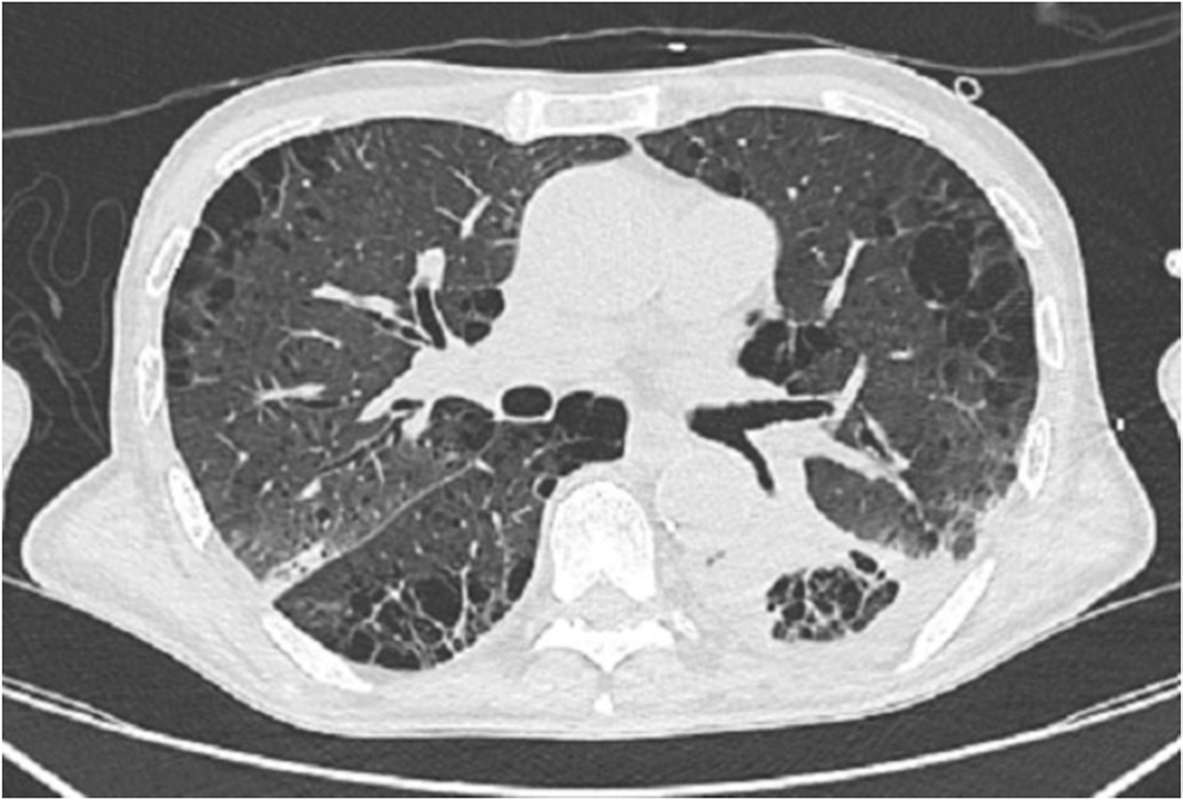
Pattern of interstitial pneumonia on chest CT scan

A total of 77-year-old patient was admitted to our university hospital in order to effort a total gastrectomy. It must be considered that the patient was affected by an adenocarcinoma on a gastric stump and had undergone a degastrogastrectomy with Roux-en-Y esophagojejunal anastomosis. This was the third stomach surgery as the patient had previously undergone gastric resection for ulcer in 1970 with Billroth II gastrojejuno anastomosis and a second resection in 1995 for an early-stage adenocarcinoma (TNM stage IA) with a new Billroth II reconstruction. About 2 months before the last operation, the patient had presented symptoms of dysphagia, vomiting, anorexia, weight loss, and anemia. Therefore, he had performed a gastroscopy and a CT scan which had ascertained the presence of a gastric adenocarcinoma. After surgery, the patient was awakened at the operating table and was admitted to the intensive care unit (ICU) for postoperative monitoring; on the second postoperative day, he returned to the general surgery ward. On the fourth postoperative day, the patient had respiratory failure with P/F ratio 150, returned to the ICU, and starts high-flow nasal cannula oxygen therapy with FiO2 40%, flow 50 l/min, and Tc 37 °C. He presented a pattern of interstitial pneumonia on chest CT scan (Fig. 1).

**Fig. 2:**
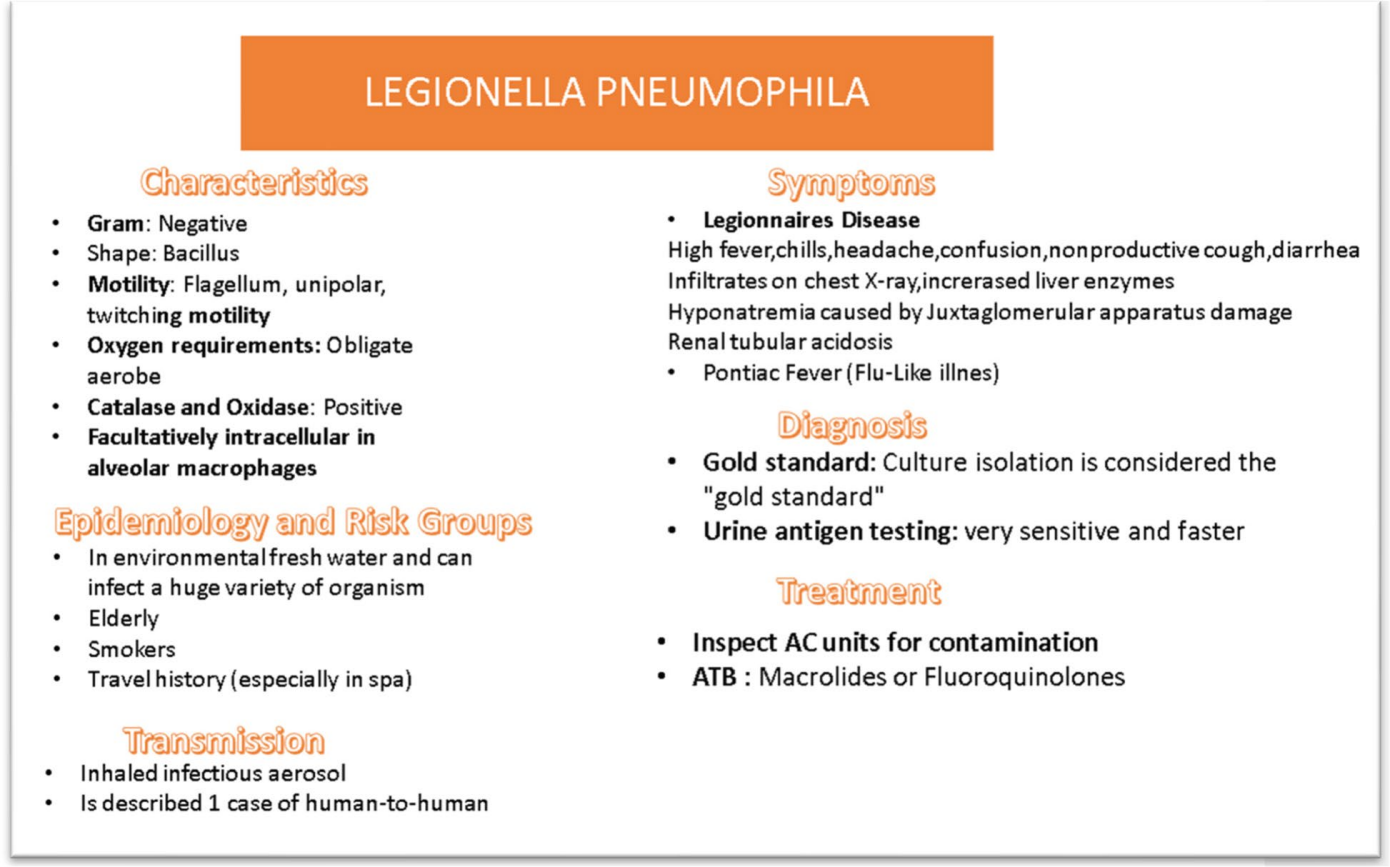
Legionella pneumophila

He undergoes a nasopharyngeal swab for SARS-CoV-2, urinary antigen research for research of *L. pneumonia* antigen. It was not possible to practice a valid bronchoaspirate and bronchial lavage as the patient was spontaneously breathing. Empiric therapy for *L. pneumophila* was started, later confirmed by urinary antigen positive for *Legionella pneumophila *(Fig. 2). Start therapy with azithromycin 500 mg every day and, on confirmation of the diagnosis, azithromycin every 12 h for 14 days.

The postoperative course was further complicated by an esophageal anastomotic leak on the seventh postoperative day, revealed by methylene blue test, and confirmed by CT scan with Gastrografin.

The surgeon indicates the placement of an endoprosthesis. To avoid the complications of mechanical ventilation in a patient affected by *L. pneumophila*, and the impossibility of maintaining a SpO2 > 92% with conventional oxygen therapy (COT) with nasal cannulas alone, we decided to continue periprocedural HFNC oxygen therapy.

We practice continuous oxygenation and ventilation during all the procedure with HFNC with the following settings: flow 60 L/min, Fio2 100%, and temperature 37 °C. Intravenous midazolam 0.05 mg/kg and sedation were started with a bolus of propofol 0.5 mg/kg and maintained in continuous infusion at 3.5 mg/kg/h. The duration of the procedure was approximately 25 min. The parameters were stable for the whole duration of the intervention with SpO2 always > 95% in spontaneous breath and MAP > 70 mmHg. After the procedure, the patient returned to the intensive care unit to complete the set antibiotic therapy. Three days after the operation, the patient finishes the 10 days of antibiotic therapy with azithromycin. During the following 10 days, we reduced respiratory support while always maintaining a P/F ratio > 250. Initially, we alternated HFNC with high FiO2 (> 40%) with MdV. From the fourth postoperative day, the patient alternated cycles of spontaneous breathing in ambient air during the day with nasal cannulas (FiO2 32%) at night. He was discharged on the 10th day without the need for chronic oxygen therapy in the surgery department.

## Discussion

*Legionella* is an aerobic, gram-negative *Bacillus*. At present, there are 58 species and over 70 serotypes of *Legionella* identified, of which at least 24 species can cause lower respiratory tract infections in humans. Approximately, 90% of *Legionella pneumonia* is caused by *Legionella pneumophila* serogroup 1, which is widely distributed in warm, humid environments and can replicate in water at 25–42 °C. Humans usually become infected with *Legionella* through inhaling *Legionella*-containing aerosols from contaminated water sources (e.g., rain, pipes, air-conditioning systems) or inhaling directly contaminated water sources in specific conditions, such as water births. After entering the respiratory tract, *Legionella* can survive and replicate exponentially in human alveolar macrophages, releasing toxins and virulence factors, resulting in *L. pneumophila* (Bai et al. 2023).

The clinical manifestations of *L. pneumophila* infections are primarily respiratory. Two quite distinct kinds of respiratory illness may result from infection; the reasons for this dichotomy are not understood. The most common presentation is acute pneumonia, which varies in severity from mild illness that does not require hospitalization (walking pneumonia) to fatal multilobar pneumonia.

Typically, patients have high, unremitting fever and cough but do not produce much sputum.

The symptoms of *Legionella* infection undoubtedly result from a combination of physical interference with oxygenation of blood, ventilation-perfusion imbalance in the remaining lung tissue, and release of toxic products from bacteria and inflammatory cells. Bacterial factors include a protease that may be responsible for tissue damage. Cellular factors include interleukin-1, which produces fever after it is released from monocytes, and tumor necrosis factor, which may be responsible for some of the systemic symptoms; it is the causal agent of 5 to 12% of sporadic community-acquired pneumonia cases. Recent studies regarding severe community-acquired pneumonia have shown that *Legionella pneumophila* is the second most common cause of admission to ICU, not far behind pneumococcal pneumonia (Winn 1996).

Gastric stump carcinoma is a clinical entity that has been known in general surgery for decades. It has been calculated that 10% of patients undergoing distal gastric resection for benign disease will develop residual gastric cancer approximately 15–20 years after the first operation, and this is primarily due to gastroduodenal reflux (Degastrogastrectomy for cancer of the gastric stump 1999). The prognosis of gastric stump cancer is generally poor, especially due to the low resectability rates associated with intra-perioperative surgical complications (Sinning et al. 2007).

Anastomotic leakage (AL) after gastrectomy is one of the most severe postoperative complications and is related to increasing mortality. Gastric cancer remains one of the most common cancers worldwide, but the mortality shows a continuously decreasing trend on account of the developments in surgical technique and perioperative management. At present, radical gastrectomy is still the only probably curative therapy for resectable gastric cancer. Nevertheless, such surgical treatment includes the standard lymph node dissection and various reconstruction methods, and this high complexity of surgical procedure leads to a high risk of death and postoperative complications. AL is a destructive and potentially life-threatening postoperative complication, which is relevant to the increasing cost for treatment, the prolongation of hospitalization, and postoperative mortality. The incidence of AL has been reported to be 1 ~ 6% in gastric cancer patients after gastrectomy. In a study conducted on 3926 patients, AL after gastrectomy risk factors were analyzed. Univariate analyses indicated that in the elderly, the low concentrations of plasma hemoglobin, albumin, and cholesterol, diabetes, tumors located in the upper third stomach, the laparoscopic approach, proximal or total gastrectomy, esophagojejunostomy, and long operation time were hte indipendent risk facots facilitating AL development. Multivariate analysis revealed that albumin concentration, diabetes, the laparoscopic approach, and proximal or total gastrectomy were the independent risk factors facilitating AL development (He et al. 2023).

High-flow nasal cannula (HFNC) oxygen is a recently developed noninvasive oxygen therapy system. It can provide heated and moist oxygen through the nasal cannula, as well as offer a much higher and predictable gas flow rate (up 60 L/min) and FiO2 (up to 100%). Studies demonstrate that HFNC completely prevents hypoxia during sedated gastroscopy via two mechanisms. First, the high-flow produces positive pressure within the nasopharyngeal space and thoracic cavity, which reduces airway obstruction and increases the end-expiratory lung volume. Second, HFNC can produce positive pharyngeal pressure during expiration with a constant flow, with the pressure mainly determined by the volume of flow and expiratory flow of the patient. Because of its potential to improve oxygenation and ventilation, HFNC has been applied in many clinical situations to prevent hypoxemia, such as in awake fiber-optic intubation, conscious sedation during bronchoscopy, and some dental treatments under intravenous sedation. In addition, a few randomized controlled trials have shown that HFNC could also reduce the risk of hypoxemia during sedated digestive endoscopy, but some studies cannot draw the same conclusion (Zhang et al. 2022).

During gastroscopy, the patient’s mouth is kept open because of the gastroscopy tube. Therefore, it is reasonable to doubt the positive airway pressure mechanism. Maintaining a constant PEEP with HFNC is challenging because it can significantly decrease with open-mouth breathing. A recent systematic review demonstrated that when subjects ventilated with HFNC opened their mouth, hypopharyngeal pressure dropped from 5.2 (3.5, 7.0) cm H_2_O to 1.1 (− 0.9, 2.4) cm H_2_O with HFNC set at 50 L/min, and nasopharyngeal pressure dropped from 6.8 to 0.8 cm H_2_O with HFNC set at 60 L/min (Li et al. 2023). The goal of this conduct is to favor the washout of CO2 in the anatomical dead space, including more distal conducting airways, and to maximize the alveolar fraction of oxygen through the replacement of nitrogen to oxygen, stored in the lungs as functional residual capacity (FRC). It results in a reduction of rebreathing of CO2, decreases the available pressure gradient for oxygen transfer to the alveolus, and hastens the onset of hypoxemia (Bartlett et al. 1959). 

We preferred to use propofol as a sleep inducer instead of a combination of midazolam and opioids due to its more predictable pharmacokinetic profile, rapid onset, and overlapping adverse effects for the dose that was used in our case.

In a recent retrospective study on procedure- and sedation-related adverse events in 73,029 endoscopies performed in the United States, Goudra et al. identified 44 patients who required endotracheal intubation and 14 deaths (Goudra et al. 2021). Therefore, reducing the incidence of hypoxia and severe hypoxia is always an important task during sedated endoscopy procedures. The optimal strategy for reducing the risks of adverse events caused by hypoxia is to prevent the development of hypoxia during the procedure.

A systematic review and meta-analysis on the effectiveness of high-flow nasal cannula during sedated digestive endoscopy demonstrated that compared to SNC (steady flow nasal cannula), HFNC not only reduce the incidence of hypoxemia but also reduce the requirements for airway interventions during sedated digestive endoscopy procedures, especially in patients at low risk for hypoxemia (Zhang et al. 2022).

## Conclusion

The management of the airways in patients affected by *Legionella* can represent a problem burdened by a series of additional risks resulting from IOT such as damage associated with mechanical ventilation, contamination of the ventilator, and delayed weaning, with an increase in hospitalization times. The use of HFNC in endoscopic procedures allows to avoid all these risks as well as the field conflict with the operator maintaining a higher oxygen saturation compared to other oxygenation devices. More studies are necessary in order to confirm this result.

## Data Availability

All of the material is owned by the authors, and/or no permissions are required. All the data and materials are after contact with the corresponding author.

## References

[CR1] Bai L, Yang W, Li Y (2023). Clinical and laboratory diagnosis of Legionella pneumonia. Diagnostics (basel).

[CR2] Bartlett RG, Brubach HF, Specht H (1959). Demonstration of aventilatory mass flow during ventilation and apnea in man. J Appl Physiol.

[CR3] Canet J, Gallart L (2013). Predicting postoperative pulmonary complications in the general population. CurrOpinAnaesthesiol.

[CR4] Degastrogastrectomy for cancer of the gastric stump. J Chir (Paris). 1999;136(3):140–4. French. PMID: 10549011 10549011

[CR5] Goudra B, Gouda G, Singh PM. Recent developments in devices used for gastrointestinal endoscopy sedation. Clin Endosc. 2021;54(2):182–192. 10.5946/ce.2020.057. Epub 2021 Mar 18. PMID: 33730777; PMCID: PMC8039741 10.5946/ce.2020.057 PMC8039741 33730777

[CR6] He Z, Liu H, Zhou L, Li Q, Wang L, Zhang D, Xu H, Xu Z (2023). Risk factors and conservative therapy outcomes of anastomotic leakage after gastrectomy: experience of 3,926 patients from a single gastric surgical unit. Front Oncol.

[CR7] Li J, Albuainain FA, Tan W (2023). The effects of flow settings during high-flow nasal cannula support for adult subjects: a systematic review. Crit Care.

[CR8] Sinning C, Schaefer N, Standop J, Hirner A, Wolff M. Gastric stump carcinoma – epidemiology and current concepts in pathogenesis and treatment,European Journal of Surgical Oncology (EJSO),2027;33(2):133–139. ISSN 0748–7983,10.1016/j.ejso.2006.09.006. 10.1016/j.ejso.2006.09.006 17071041

[CR9] Smetana GW, Lawrence VA, Corlell JE; American College of Physicians. perioperative pulmonary risk stratification for noncardiothoracic surgery: systematic review for the American College of Physicians. Ann Intern Med. 2006;144(8):581–95. 10.7326/0003-4819-144-8-200604180-00009. PMID: 16618956 10.7326/0003-4819-144-8-200604180-00009 16618956

[CR10] Winn WC Jr. Legionella. In: Baron S, editor. Medical Microbiology. 4th edition. Galveston (TX): University of Texas Medical Branch at Galveston; 1996. Chapter 40. Available from: https://www.ncbi.nlm.nih.gov/books/NBK7619/

[CR11] Zhang YX, He XX, Chen YP, Yang S (2022). The effectiveness of high-flow nasal cannula during sedated digestive endoscopy: a systematic review and meta-analysis. Eur J Med Res.

